# Prevalence and associated factors of stunting, wasting and underweight of children below five using quintile regression analysis (PDHS 2017–2018)

**DOI:** 10.1038/s41598-022-24063-2

**Published:** 2022-11-25

**Authors:** Maryam Siddiqa, Amber Zubair, Asifa Kamal, Muhammad Ijaz, Tahani Abushal

**Affiliations:** 1 Department of Mathematics and Statistics, International Islamic University Islamabad, Islamabad, Pakistan; 2 Department of Statistics, Lahore College for Women University, Lahore, Pakistan; 3 Department of Mathematics and Statistics, The University of Haripur, Haripur, KP Pakistan; 4 Department of Mathematical Sciences, Umm Al-Qura University, Mecca, Saudi Arabia

**Keywords:** Risk factors, Mathematics and computing

## Abstract

The objective of the current study is to identify the risk factors for malnutrition among the age of under-five children’s in Pakistan. This is secondary data analysis for the data taken from Pakistan Demographic and Health Survey (PDHS 2017–18) and was analyzed by implementing quantile regression analysis. The sample size included 12,708 alive children in the study, for which the data collection period was from November 22, 2017, to April 30, 2018. The prevalence of malnutrition among boys is high (51.2%). Older age mother’s children have more prevalence of malnutrition (20.7%). A child born with small body size (underweight: Q_0.25_: − 0.625; Q_0.50_: − 0.623; Q_0.75_: − 0.426 and wasting: Q_0.50_: − 0.513); having uneducated mother (underweight: Q_0.25_: − 0.387; Q_0.50_: − 0.247; Q_0.75_: − 0.328), belonged to a poor household (underweight: Q_0.50_: − 0.251),residing in rural areas (underweight: Q_0.25_: − 0.443), not following properly breastfeeding practices (underweight: Q_0.50_: − 0.439; Q_0.75_: − 0.438) have negative effect on different measures of malnutrition and this effect is significantly raises across different quantiles of stunting , wasting and underweight (at *p value* < 0.01 and < 0.05). Older age mother (stunting: Q_0.50_: 0.777; Q_0.75_: 1.078; underweight Q_0.20_: 0.568; Q_0.50_: 0.429; Q_0.75_: 0.524) and higher birth order number (stunting: Q_0.50_: 0.415; Q_0.75_: 0.535), have a positive effect on three measures of under-nutrition and this effect is gradual raises at different quantile of stunting, wasting and underweight. Elder and smoker mothers were proved associated risk factors of both stunting and being underweight in Pakistan. Moreover, Proper breastfeeding practices, better economic status, average or above the average birth weight of the child, and milk consumption are found protective factors against stunting, wasting, and underweight children in Pakistan.

## Introduction

Malnutrition is one of the severe health problems that prevail in developing countries and it contributes significantly to child mortality^1,2^. It is referred to as ill health caused by nutritional deficiencies of calories, vitamins, and minerals, protein, interrelating with infections, and other poor well-being and social conditions^3^. Proper nourishment is essential for a human that remains unmet for massive numbers of children in several developing countries^3^. Poor nutritional status of children has a close link with general standards of living and underpins both child mortality and poverty^3^.

Severe consequences of being malnourished through childhood are related to impeding behavior, delayed mental, and physical growth^3^. These delayed cognitive and motor developments can diminish the academic performance and social skills of children and in long term can increase the risk of developing ailments or disabilities and even death^3^. Malnutrition has both short and long-term determinable adverse health effects and these are caused by various interlinked factors^4^. It has also an impact on children’s cognitive and physical development. It increases the risk of infections that leads to child mortality and morbidity^4^.

Malnutrition is a silent and invisible emergency^5^. It is the reason for nearly 5.2 million under 5 children deaths each year in developing countries^6^. Particularly South Asian countries suffer from a high child malnutrition rate referred to as Asian Enigma^7^. In the second international conference on nutrition (ICN2), United Nations (UN) declared the decade (2016–2025) as a decade of action to accomplish the tasks set for global nutrition and also to reach SDG2, and SDG3 targets relevant to malnutrition^8^. Globally, children under age 5 are found at higher risk of malnutrition as compared to other age groups. Almost half (45%) of child mortality is associated with undernutrition^8^. By 2020, 149 and 45 million children in the world were estimated as stunted and wasted, respectively^8^.

After 2000, stunting reduced substantially all over the world, but the decline needs to be accelerated to attain the 2030 target. Wasting is still prevalent globally at an alarming rate^9^.

In Pakistan, among children less than five years, 44% were found stunted, 31% were found underweight and 15% were found wasted^4^. In addition, according to the National Nutrition Survey (2018) of Pakistan, 40% of children under five years of age were found stunted, 17.7% were wasted and nearly one-third of children were reported underweight (28.9%)^5^. Under-nutrition is common in all age groups of children of Pakistan^10,11^. According to publications of the State Bank of Pakistan, approximately half of under 5 children are stunted in Pakistan and one child out of 10 suffered from wasting^12^. Pakistan is among those seven countries of the world that bear the burden of 2/3 of the undernourished population of the world^12,13^. Globally, Pakistan has been placed at 77th number out of 113 countries that are facing malnutrition. In the South Asian Region, Pakistan has the highest number of stunted children while if a comparison is made for southern countries, Pakistan has the highest wasting of children^14–16^.

The prevalence of stunting declined from 1965 to 1994 from 48 to 36.3% but the situation got deteriorated from 2001 to 2011i.e. percentage stunted increased from 41.6 to 43.7%^5^. It is still at a global critical level of 40.2% in 2018^5^. The annual reduction rate is predicted to be 0.5%, which is insufficient to get a significant decline in the stunting rate in Pakistan^5^. The prevalence of wasting also increased from 1997 to 2011 from 8.6 to 15.1%. In 2018, the prevalence of wasting has risen to 17.7%^5^. According to (PDHS 2017–18) report, from 2012–13 to 2017–18, the prevalence of stunted decreased from 45 to 38%. A similar trend of wasting and being underweight was also observed. Underweight and wasting declined from 30 to 23% and 11 to 7% respectively from 2012–13 to 2017–18^17^. A decline in the percentage of underweight, stunted, and wasted children in the last decade is at a slow pace to attain a target of SDG 2.2 i.e. to end malnutrition by 2025 and needs to be addressed by the researcher to explore the factors behind it. Public health researchers must identify factors that are creating hurdles in attaining SDG 2.2 target for Pakistan and consequently suggest policy maker’s effective, evidence-based recommendations^17^. Pakistan National Nutrition Survey^5^ reported insufficient diet, frequent infections, poor breastfeeding practices, delayed introduction of complementary foods, and inadequate protein in the diet as the causes of childhood poor nutrition^5^. It is considered in a clinical perspective as well as in a bio-social context, as several socio-demographic factors lead to malnutrition^5^. Sand et al.^18^ revealed that mother literacy, low income, and overcrowding are significant factors of stunting in the Province of Sindh-Pakistan. Existing literature showed that body mass index, birth order number, rural settlement, mother education, and wealth index are also significantly associated with malnutrition^19,20^.

Malnutrition is a treatable condition with prompt identification and prevention. For prompt identification and prevention, it is important to identify factors associated with it. This study contributes to understanding the effect of societal, maternal, and child level factors on the stunting, wasting, and underweight among under 5 children in Pakistan at different quantiles of these three measures of malnutrition. In the current study, a recent wave of authentic data set PDHS 2017–18 has been used.

## Methods

The secondary-data analysis study was conducted by extracting data from Pakistan Demographic and Health Survey (PDHS) 2017–18 for which the data-collection period was from November 22, 2017, to April 30, 2018^17^. It is the fourth and up till the last round of conducted Survey. All the experimental protocols were approved by the institutional ethical review committee of Islamic International University Islamabad, Pakistan. Moreover, it is also confirming that all methods were carried out in accordance with relevant guidelines and regulations.

PDHS surveys involve the complex sampling procedure and standardized techniques in their survey for the selection of samples from rural and urban areas of 8 regions of Pakistan. A two-stage stratified clustered sampling design was followed by using a sampling frame prepared by the Pakistan Bureau of Statistics. Through a two-stage selection process, samples were selected independently in each stratum. There were 16 sample strata in total. In the 1^st^ stage, a cluster was selected based on probability proportional to size consisting of enumeration blocks (EBs). A total of 580 primary sampling units were selected (295 rural and 285 Urban). In each of the selected clusters, a complete list of the household was obtained. The list of households served as the sampling frame for the selection of households to be interviewed in the second stage. In the second stage, systematic sampling with equal probability was used in selecting the specified numbers of households in each cluster of the interview, and 28 per cluster were selected. In the PDHS sample, PSUs are villages/mouzas for rural areas and enumeration blocks for urban areas. The total sample size of alive children comprised 12,708 from PDHS (2017–18)^17^. In the current analysis, 15,068 ever-married women of 8 regions of Pakistan were interviewed about the malnutrition status of their children.

Explanatory variables comprised child-level variables, maternal variables, and societal variables. These variables include sex of child (male, female), child size at birth (small, average and, large), birth order number, Baby postnatal check-up (no, yes), Consumed Fresh milk (no, yes), Consumed formula milk (no, yes), Breastfed (no, yes), Initiation of Breastfeeding (Immediately, within 1st hour and 1st day), Child age in months, Mother education (no education, primary and secondary and higher), Mother age, Maternal BMI (underweight, normal and overweight and obese), Mother working status (not working, working), During pregnancy usage of iron tablets (no, yes), Smoke cigarettes (no, yes), Region (Punjab, Sindh, KPK, Baluchistan, GB, ICT, AJK and FATA), Type of place of residence (urban, rural), wealth index (poor economic status, middle-class economic status and rich economic status), Father education (no education, primary and secondary and higher).

Among several ways of measurement of the nutritional status of a child, anthropometric measurements based on height and weight ratios are most commonly used to assess whether the child is likely to be properly nourished, undernourished, or over-nourished^4,21–23^. These measurements of the nutritional status are referred to as height-for-age HAZ score (stunting), weight-for-height WHZ score (wasting) and weight-for-age WAZ score (underweight). Stunting, wasting, and underweight scores are three response variables of the current study. Compared with reference data collected by the Pakistan Demographic Health Survey Report (PDHS, 2017–18)^17^, deficiencies in any of the anthropometric indicator is considered evidence of malnutrition and denoted as stunting, wasting, and underweight, respectively. Generally, the prevalence of undernourished children is calculated as the ratio of children who are less than − 2 standard deviation of z-score, lying down the median of the international reference population (PDHS report 2017–18)^17^.

Quantile regression utilized the idea of conditional quantile function^24^. It is the extension of linear regression when conditions of ordinary least square (OLS) regression like homoscedasticity and normality are not satisfied. For this purpose, we assessed heteroscedasticity by using Breusch-Pagan/Cook-Weisberg test for HAZ score stunting (Chi^2^: 644.89, P value < 0.001), for WHZ score wasting (Chi^2^: 509.37, P value < 0.001), for WAZ score underweight (Chi^2^: 896.66, P value < 0.001) and significant P values show the fact that covariates are not homogeneous across the distribution of response variables are heterogeneous and their effect may differ across the quantiles of three response variables. Statistical test Shapiro–wilk W test of normality was assessed, for HAZ score stunting (W: 0.195, P value < 0.001), for WHZ score wasting (W: 0.173, P value < 0.001), for WAZ score underweight (W: 0.173, P value < 0.001) and significant P values show the distribution of response variables are not normal. In this case, the Quantile regression model is the more robust method. The quantile regression (QR) model was used to examine the patterns of risk factors effects at different points of the conditional distribution of the measures of malnutrition (stunting, wasting, and underweight). QR provides a more complete picture of the impact of independent variables on the entire distribution of response variables measures of malnutrition by modelling the conditional median of independent variables^24^. Coefficients of ordinary least squares (OLS) and quantile regression (QR) were plotted using a facet plot. It best depicts the change in the level and trend of coefficients of OLS and QR models. It can be observed from Supplementary figures S1, S2, and S3 that the effect of factors varies for lower, median, and upper quartile regression for the three models of stunting, wasting, and underweight. That reflects the difference in trend at different quantile regression, which means variables have varying effects at different levels of the conditional distribution. The OLS model is not sufficient enough to reflect the effect of various factors on stunting, wasting, and underweight and hence necessitate fitting QR models to study how the effect varies at different points of the conditional distribution of response variables.

Moreover, by plotting Cook’s D plot (Supplementary figures S4, S5, S6) we have verified the presence of outliers in the data set. So Quantile regression is more robust to outliers than OLS, as we do not need to make strong stochastic assumptions in quantile regressions (See Cameron and Trivedi, 2005, p. 85)^24^. All statistical analysis was done by using STATA (Version 13).

## Results

Descriptive statistics of three response variables are presented in Table 1. Stunting, wasting, and being underweight are the measures of malnutrition.

**Table 1:** Descriptive statistics of response variables.

Variables	Mean(S.D)	Min	Max
Stunting (HAZ)	0.492 (14.353)	− 5.99	99.98
Wasting(WHZ)	1.561 (13.380)	− 4.94	99.98
Underweight(WAZ)	− 0.929 (4.567)	− 5.99	99.98

Descriptive statistics of a child, mother, and societal characteristics that affect the measures of child malnutrition are presented in Table 2.

**Table 2:** Descriptive statistics of explanatory variables—child, maternal and societal characteristics for malnutrition—Pakistan Demographic Health Survey (2017–2018).

Variables	Variable attributes	Frequency	Percentage
Sex of child	Male	2145	50.8
	Female	2081	49.2
Size of the child at birth	Small (< 45.7 cm)	725	17.2
	Average (45.7–60 cm)	3183	75.3
	Large (> 60 cm)	317	7.50
Baby postnatal checkup	No	3061	75.7
	Yes	981	24.3
Consumed fresh milk	No	2947	70.7
	Yes	1223	29.3
Consumed formula milk	No	3018	71.4
	Yes	1206	28.6
Breast fed	No	1904	45.1
	Yes	2322	54.9
Initiation of breastfeeding	Immediately	2052	50.7
	Within 1st hour	958	23.7
	Within 1 day	1040	25.7
Mother’s education	No education	2175	51.5
	Primary	579	13.7
	Secondary and higher	1472	34.8
Maternal BMI	Underweight	361	8.54
	Normal	1919	45.4
	Overweight and obese	1946	46.0
Mother working status	Not-working	3675	87
	Working	551	13
During pregnancy usage of iron tablets	No	1483	35.2
	Yes	2735	64.8
Smoke cigarettes	No	4079	96.5
	Yes	145	3.43
Region	Punjab	906	21.4
	Sindh	806	19.1
	KPK	702	16.6
	Baluchistan	513	12.1
	GB	278	6.58
	ICT	234	5.54
	AJK	436	10.3
	FATA	351	8.3
Type of place of residence	Urban	1926	45.6
	Rural	2300	54.4
Economic status (wealth index)	Poor economic status	1956	46.3
	Middle class economic status	805	19.0
	Rich economic status	1465	34.7
Father’s education	No education	1157	27.4
	Primary	620	14.7
	Secondary and higher	2397	56.7
**Continuous variables**			
Variables	Frequency	Mean (S.D)	
Child age	4226	3.304 (0.0141)	
Birth order number	4226	1.762 (0.0039)	
Mother age	4226	1.970 (0.0015)	

*KPK* Khyber Pakhtunkhwa, *GB* Gilgit Baltistan, *ICT* Islamabad Capital Territory, *AJK* Azad Jammu and Kashmir, *FATA* Federally Administered Tribal Area.

Table 2 provides descriptive statistics for child-level, maternal and societal characteristics. Half of the children (50.8%) were males and the majority of children (75.3%) were average-sized at birth. The majority of children (75.7%) did not receive a baby postnatal check-up and (70.7%) did not consume fresh milk. Also, 54.9% of children were reported being breastfed and for 50.7% of children, breastfeeding was initiated immediately after birth. More than half (51.5%) of mothers had no formal education, 46.0% were overweight or obese and 8.54% were underweight. Only a small segment of mothers (13.0%) for formally employed whereas 87.0% were not employed in salaried jobs. Women were predominantly non-smokers (96.5%). Among societal-level factors, 45.6% of the children belonged to families that reside in urban areas. Around 46.3% of the children belonged to families with poor economic status. The fathers of 56.7% of the children had secondary and higher-level education.

Table 3 shows the quantile regression estimates for modelling Stunting, Wasting and Underweight-anthropometric measures of malnutrition.

**Table 3:** Quantile regression estimates for factors associated with stunting (HAZ), underweight (WAZ), and wasting (WHZ) below five years of age—PDHS (2017–2018).

Variables	Stunting (HAZ)			Underweight (WAZ)			Wasting (WHZ)		
	Q_0.25_	Q_0.50_	Q_0.75_	Q_0.25_	Q_0.50_	Q_0.75_	Q_0.25_	Q_0.50_	Q_0.75_
Place of residence (urban* vs. rural)	0.428 (0.07)	0.087 (0.70)	− 0.170 (0.54)	0.443** (< **0.001**)	0.269 (0.08)	0.122 (0.57)	0.310 (0.27)	0.101 (0.58)	− 0.134 (0.57)
Father education (no education* vs. primary, secondary and higher)	0.055 (0.68)	0.226 (0.18)	0.311 (0.11)	− 0.117 (0.34)	0.071 (0.37)	− 0.015 (0.88)	− 0.082 (0.58)	0.075 (0.43)	− 0.101 (0.28)
Mother education (no education* vs. primary, secondary and higher)	0.300 (0.13)	0.146 (0.26)	0.021 (0.90)	− 0.387** (< **0.001**)	− 0.247* (**0.03**)	− 0.328** (< **0.001**)	0.171 (0.26)	0.126 (0.12)	0.150 (0.12)
Mother working status (not working* vs. working)	0.008 (0.98)	0.016 (0.95)	− 0.019 (0.96)	− 0.175 (0.44)	− 0.08 (0.70)	− 0.085 (0.71)	− 0.165 (0.71)	0.139 (0.55)	− 0.124 (0.66)
Sex of child (male* vs. female)	0.262 (0.23)	0.257 (0.23)	0.067 (0.81)	− 0.043 (0.76)	0.108 (0.39)	− 0.006 (0.96)	− 0.219 (0.41)	− 0.077 (0.62)	− 0.009 (0.95)
Mother age	0.416 (0.07)	0.777** (< **0.001**)	1.078** (< **0.001)**	0.568** (**0.01**)	0.429** (< **0.001**)	0.524* (**0.01**)	0.135 (0.64)	0.031 (0.90)	0.388 (0.16)
Birth order number	− 0.141 (0.46)	0.415* (**0.02**)	0.535** (< **0.001)**	− 0.026 (0.85)	− 0.147 (0.17)	− 0.151 (0.21)	0.066 (0.75)	− 0.114 (0.44)	2.333 (0.01)
Smoke cigarettes (no* vs. yes)	1.555 (0.10)	1.856* (**0.028**)	1.097 (0.98)	− 0.156 (0.85)	0.311 (0.74)	1.259** (< **0.001**)	0.527 (0.64)	0.646 (0.56)	0.750 (0.97)
During pregnancy usage of iron tablets(no* vs. yes)	0.178 (0.49)	− 0.147 (0.59)	− 0.385 (0.13)	0.009 (0.95)	− 0.197 (0.16)	− 0.061 (0.71)	− 0.149 (0.55)	− 0.212 (0.25)	− 0.091 (0.57)
Baby postnatal checkup(no* vs. yes)	− 0.101 (0.72)	− 0.021 (0.93)	0.122 (0.68)	− 0.034 (0.87)	− 0.318 (0.11)	0.002 (0.99)	− 0.321 (0.91)	0.020 (0.91)	− 0.080 (0.70)
Consumed fresh milk (no* vs. yes)	− 0.201 (0.50)	− 0.259 (0.31)	0.083 (0.73)	0.101 (0.62)	0.070 (0.69)	− 0.099 (0.58)	0.053 (0.81)	0.172 (0.39)	− 0.132 (0.51)
Consumed formula milk (no* vs. yes)	− 0.167 (0.66)	0.090 (0.78)	0.243 (0.46)	− 0.433 (0.32)	− 0.228 (0.37)	− 0.428** (< **0.001**)	− 0.167 (0.56)	− 0.832** (< **0.001**)	− 0.516 (0.08)
Wealth index (poor economic status* vs. middle class economic status, rich economic status)	0.273 (0.32)	− 0.013 (0.94)	− 0.265 (0.12)	− 0.251** (**0.03**)	0.218 (0.16)	0.018 (0.83)	0.293 (0.24)	0.006 (0.67)	0.537 (0.71)
Breastfed (no* vs. yes)	− 0.473 (0.12)	− 0.223 (0.54)	− 0.176 (0.58)	− 0.400 (0.20)	− 0.439* (**0.02**)	− 0.438* (**0.03**)	− 0.280 (0.39)	− 0.210 (0.38)	0.232 (0.24)
Initiation of breastfeeding (immediately* vs. within 1st hour, within 1st day)	0.141 (0.19)	0.189 (0.09)	0.140 (0.14)	0.093 (0.36)	0.152 (0.05)	0.164* (**0.04**)	− 0.035 (0.72)	− 0.091 (0.35)	− 0.093 (0.27)
Maternal BMI (underweight* vs. normal, overweight and obese)	0.262 (0.19)	0.266 (0.11)	0.310 (0.17)	0.089 (0.55)	0.117 (0.45)	0.123 (0.41)	− 0.069 (0.66)	0.044 (0.73)	0.180 (0.29)
Size of child at birth (small* vs. average, large)	0.333 (0.19)	0.209 (0.31)	0.244 (0.32)	− 0.625** (< **0.001**)	− 0.623** (< **0.001**)	− 0.426** (< **0.001**)	0.392 (0.11)	− 0.513** (< **0.001**)	0.405 (0.103)
Child age in months	0.595 (0.21)	0.589 (0.20)	0.636 (0.43)	− 0.128 (0.33)	0.293 (0.12)	0.427 (0.20)	0.107 (0.41)	− 0.093 (0.40)	− 0.430 (0.60)

**Indicate at 0.01, *indicate at 0.05, significant p values shown in bold. * indicating reference category.

The Quantile regression model of stunting shows that the variables mother’s age, birth order number, and smoking status of mother have a significant positive impact on stunting. The result of the Quantile regression model indicates that one year increase in the mother’s age is positively associated with stunting and it has a 0.777 unit increase in stunting at the 50th quantile and a 1.078 unit increase in stunting at the 75th quantile. This positive effect on stunting gradually increases at the 50th and 75th quantile High birth order is positively associated with stunting and it increased to 0.415 units in stunting at the 50th quantile and 0.535 units increased in stunting at the 75th quantile. Smoker mother’s have also a positive effect on stunting in children at the 50th quantile (1.856) as compared to non-smoker mothers.

Mother’s age, mother’s smoking status, initiation of breastfeeding, and type of place of residence (rural) have a positive impact on underweight while mother’s education, breastfeeding, consumption of formula milk, size of child at birth, and wealth index are negatively associated with underweight. One year increase in the mother’s age is positively associated with being underweight. The positive effect considerably gradually increases at the lower and upper quantiles. It is increased by 0.568 units in underweight at the lower quantile, 0.429 unit increases in underweight at the 50th quantile and 0.524 unit increases in underweight in upper quantile for per year increase in maternal age. Smoker mother’s have significant positive impacts on the underweight and it is increased at 75th quantile (1.259) for smoker mothers as compared to nonsmokers mothers). Late initiation of breastfeeding is also associated with underweight and it is increased at 75th quantile (0.164) for mothers who initiated breastfeeding late as compared to their other counterparts The effect of rural settings on underweight children is large and increased in underweight at 25th quantile (0.443) as compared to urban residents. Mother’s education is inversely associated with underweight and the effect of the mother education on underweight is monotonically decreasing at 25th quantile (0.387) and 75th quantile (0.328) as compared to 50th quantile (0.247) for children born to educated mothers as compared to uneducated mothers. Breastfeeding is inversely associated with underweight and it is found that the decrease in underweight at the 50th quantile is 0.439and 0.438 decreases in upper quantile for children who were breastfed as compared to those who were not breastfed. The large size of a child at birth had a negative effect on the underweight and this effect is gradually raising and being underweight is decreased by 0.625 at lower quantile, 0.623 at 50th quantile, and 0.426 at upper quantile respectively as compared to a child born small in size. Rich economic status is significantly negatively associated with underweight and there was a decrease of 0.251 in underweight at the 25th quantile for a child born to a rich household as compared to a poor household. Consumption of formula milk is significantly inversely associated with underweight and 0.428) decreased in underweight at upper quantile for those children who consumed formula milk as compared to those who had not use it.

The results indicated that the size of a child at birth and consumption of formula milk is inversely associated with wasting. The large size of a child at birth is significantly negatively associated with wasting and wasting is reduced by 0.513 at the 50th quantile as compared to the small birth size of a child. Consumption of formula milk is also significantly inversely associated with wasting and it reduced wasting by 0.832 at the 50th quantile as compared to those children who did not consume it.

## Discussion

In this paper, first, we investigate the determinants of stunting, wasting, and underweight by using the Quantile Regression technique to see how societal, maternal and child level factors affect the measures of malnutrition stunting, wasting and underweight at different quantiles of the distribution of response variables. Malnutrition is considered one of the most significant problems in less privileged countries. Identification of determinants of malnutrition in the specific community is essential for the implementation of preventive and control measures to reduce the burden of malnourished children. The south Asian sub-region is facing a serious malnutrition burden among children of age below 5 years. The prevalence of stunting in Pakistan is 31.7%, and the prevalence of wasting is 14.3%, which is significantly higher than the global average of 21.3% and 6.9%, respectively^5^.

Quantile Regression results suggest that the most important factors for the improvement of child health are maternal factors and published literature emphasized the positive impact of improved maternal education on child’s health^4,25,26^. Smoker and older mothers are observed at higher risk of having stunting or underweight children. Children who had higher birth orders have more risk of stunting in the future. Children living in rural areas were also observed to be underweight as compared to urban children. Rich children had less risk to be stunted as compared to poor children. Children of educated mothers were found at lesser risk of being underweight as compared to uneducated mothers. Moreover, use of formula milk, children who were breastfed and children who immediately started breastfeeding are observed protected against underweight problems. The use of formula milk also provides children a shield against wasting. Children born with average or above average weight are observed to be not underweight or wasted as compared to children born of small size.

The highest birth order number is associated with a higher risk of stunting. Our results are consistent with several previous studies^21,27^. On the other hand, our result is contradicting with the study^28^ conducted at Bahawalpur, Pakistan which concluded no significant association was observed between birth order number and the probability of being stunted and wasted.

Children residing in rural areas are more liable to be underweight. Our results are in line with numerous studies^22,29^. Generally, the rural areas have lack primary health care services, clean water supply, proper housing and adequate hygiene facilities, which are necessities for sufficient nutrition as well as for desired growth of children. Our findings are not consistent with study^29^ conducted on the earlier wave of PDHS in which it was concluded that there is no association between stunting, wasting and underweight and sort of place of home.

Childbirth size is inversely associated with wasting and underweight in our findings. Children perceived large size at birth had a lower risk of wasting and underweight. These findings are in line with other existing studies^4,22,30^ that reported children with small birth sizes were more liable to be wasting and being underweight. The possibility of the mall birth size of a child could be due to poor maternal nutrition in the course of pregnancy, where the child is entirely dependent on the mother for nutrition in utero via the placenta, and deficiency of nutrients from the mother will adversely affect the fetus growth and development.

A Mother’s educational statu**s** plays an important role in the nutritional status of children. There exists an inverse relationship between mother education and a child undernutrition. Our research support the explanations of several studies^1,23,24^ Owing to the fact that educated mothers are more empowered and have enough health care awareness to take care of their children, over and above, educated mothers are more conscious about their children’s health.

Wealth status was found as the most significant predictor of child malnutrition which indicates their economically disadvantaged families. Children born to rich families are less liable to be Underweight. Our finding is consistent with the previous studies stated that the children from middle-class and rich families were less liable to be underweight as compared to the children of poor families^1,25,29^. Affordability or access to healthy food is working behind this phenomenon. While the study conducted in Bahawalpur concluded that there is no significant association between under-nutrition (stunting, wasting, and underweight) and wealth index^28^.

Children who were breastfed were less likely to be underweight. Our findings endorsed the previous studies^21,31,32^ that breastfeeding prevents a child from being underweight but if the duration of breastfeeding is long it can lead to an increase in underweight because breast milk without complementary food is not sufficient to fill the developing infant’s micronutrient needs^25^. Above six months’ children cannot rely solely on breastfeeding and require additional supplementary food for proper growth. Specifically, the rural areas require special consideration in child health programs for the decision making of the mothers to discontinue the mother-feed at an appropriate time and also start supplementary food along with breastfeeding. The estimated prevalence of exclusively breastfed infants aged 0 to 5 months is 57.2% in the Southern Asia sub-region, which is considerably greater than the global average of 44.0% Pakistan National Nutrition Survey^5^.

An increased in the mother age is associated with a current study supported by existing literature^33^. On the contrary, teenage mothers were also more likely to be malnourished^34^. Smoker mother’s children were more likely to be malnourished (stunting and underweight) in our study.

Late initiation of breastfeeding is associated with higher chances of under-nourished in terms of being underweight. Early initiation of breastfeeding is associated with lower odds of being underweight^35,36^. The beginning of breastfeeding within one hour after birth is recommended at present, for a better children’s nutritional status. With mother feeding children receive colostrum which reaches immunoglobulin and other bioactive molecules, involving developmental factor which is necessary for nutrition, growth and healthy growth of children^37^.

Consumption of formula milk is associated with a lower risk of being malnourished in form of wasting and underweight. Consumption of formula milk is associated with a reduced probability of being malnourished in the absence of breast milk^38^.

## Conclusion and recommendations

Smoking and the older age of the mother were found risk factors for both stunting and being underweight in Pakistan. Breastfeeding practices, high economic status, average or above the average birth weight of the child, and use of formula milk are protective factors against stunting, wasting, or underweight children. Awareness campaigns should be launched to prevent mothers from smoking and to promote breastfeeding. Older mothers should be provided supplements during pregnancy. Pregnant women should be careful about their dietary intake during pregnancy for the health outcomes of pregnancy.

## Supplementary Information

Supplementary Figure 1.

Supplementary Figure 2.

Supplementary Figure 3.

Supplementary Figure 4.

Supplementary Figure 5.

Supplementary Figure 6.

## Data Availability

The data set used in the study taken from the Pakistan Demographic and Health Survey (PDHS) website and the files are available at the following url: https://dhsprogram.com/data/dataset/Pakistan_Standard-DHS_2017.cfm?flag=1.
