# Supplementary material for: Prevalence and associated factors of stunting, wasting and underweight of children below five using quintile regression analysis (PDHS 2017–2018)
Source: Sci Rep. 2022 Nov 25;12:20326. doi: 10.1038/s41598-022-24063-2 (PMC9700674; doi:10.1038/s41598-022-24063-2)
Supplement: Supplementary file 6 — Supplementary Figure 6. [file 41598_2022_24063_MOESM6_ESM.docx]

**Figure 6. Graphical representation of Outliers by using COOK’S D FOR UNDERWEIGHT (WAZ)**
